# Antibiotic Residues in Milk as a Consequence of Mastitis Treatment: Balancing Animal Welfare and One Health Risks

**DOI:** 10.3390/vetsci12121159

**Published:** 2025-12-04

**Authors:** Dragana Tomanić, Nebojša Kladar, Zorana Kovačević

**Affiliations:** 1 Institute of Food Technology in Novi Sad, University of Novi Sad, 21000 Novi Sad, Serbia; 2 Department of Pharmacy, Faculty of Medicine, University of Novi Sad, 21000 Novi Sad, Serbia; 3 Centre for Medical and Pharmaceutical Investigations and Quality Control (CEMPhIC), Faculty of Medicine, University of Novi Sad, 21000 Novi Sad, Serbia; 4 Department of Veterinary Medicine, Faculty of Agriculture, University of Novi Sad, 21000 Novi Sad, Serbia

**Keywords:** bovine mastitis, animal welfare, antibiotic residues, antimicrobial resistance, one health

## Abstract

Bovine mastitis is a prevalent infectious disease in dairy cattle, causing inflammation, pain, reduced milk yield, and economic losses. Antibiotic therapy is the mainstay of treatment, yet irresponsible use can lead to the presence of antibiotic residues in milk and contribute to the emergence of antimicrobial resistance (AMR), posing significant risks to public health and food safety. This review aims to provide a comprehensive synthesis of current knowledge on mastitis management, antibiotic use and resulting residues in milk, their public health and environmental impacts, and alternative strategies to reduce antibiotic dependence, framed within a One Health–One Welfare perspective. Antibiotic residues in milk are closely linked to treatment practices, withdrawal period compliance, and regulatory oversight, with prevalence ranging from <1% in some European countries to over 80% in parts of Africa. Residues, particularly from β-lactams, tetracyclines, and quinolones, can disrupt human intestinal microbiota, promote resistant bacterial strains, trigger immunological reactions, and interfere with dairy processing. Environmental contamination through excreted antibiotics further facilitates the spread of resistance. Sustainable alternatives, including probiotics, phytotherapy, vaccines, and improved farm biosecurity, show promise in reducing antibiotic use while maintaining animal welfare and productivity. Antibiotic therapy remains essential for mastitis control, but its consequences on milk safety, public health, and AMR require prudent management. Integrating monitoring, adherence to withdrawal periods, and sustainable alternatives within a One Health–One Welfare framework is critical for ensuring safe, responsible, and environmentally sustainable dairy production.

## Simple Summary

Bovine mastitis is one of the most common diseases in dairy cows, causing pain, reduced milk production, and economic losses. Antibiotics are widely used to treat and prevent mastitis, improving animal welfare, but their use can lead to residues in milk. These residues may pose risks to human health, including antibiotic resistance, allergic reactions, and microbiota disruption. This review highlights the balance between maintaining animal welfare and minimizing public health risks. It also explores sustainable alternatives, such as probiotics, vaccines, biosecurity measures, and plant-based therapies that can reduce reliance on antibiotics while safeguarding both animal and human health. Integrating these strategies within a One Health framework is essential for safe, sustainable dairy production.

## 1. Introduction

The dairy industry is a cornerstone of global agriculture, providing milk and dairy products that are both nutritionally essential and of major social and economic importance worldwide [1]. It accounts for approximately 15% of total agricultural production [2], with cow’s milk alone representing 81% of global output [3]. With over six billion consumers, dairy is integral to the global food economy, and per capita consumption is projected to grow by 2030 at a rate of 1% annually [2,4].

However, the dairy sector faces multiple challenges, among which bovine mastitis stands out as a major infectious disease of the mammary gland that causes inflammation, pain, and reduced milk yield, with significant consequences for both animal welfare and farm profitability [5,6]. Beyond its economic and public health dimensions, mastitis is a critical welfare concern, as affected cows experience pain, fever, reduced feed intake, and social withdrawal, all of which compromise their well-being [7]. Framing mastitis and antibiotic use within a broader One Health–One Welfare perspective is therefore essential to fully capture their multidimensional impacts [8]. Antibiotics remain the mainstay of mastitis prevention and treatment, safeguarding herd health and maintaining productivity. However, their irresponsible use also leads to undesirable consequences, most notably the presence of antibiotic residues in milk and the development of antimicrobial resistance (AMR) [9,10]. Indeed, antibiotics represent the most frequently detected veterinary drug residues in food, with reported prevalence ranging from less than 1% in Europe—where most detections are within the maximum residue limits (MRLs) established by legislation—to over 90% in some African regions, where exceedances of these limits are more frequently reported [11,12].

Globally, the total amount of antibiotics used in livestock is estimated to be approximately twice that used in humans, with 80–90% of food-producing animals receiving antibiotic treatment at some point during their lifetime [13,14,15,16]. Annual antibiotics consumption in livestock is estimated at 63,000 tons and is expected to increase by 70% by 2030 [17]. While the use of antibiotics as growth promoters was banned in the European Union (EU) in 2006, such practices remain common in several regions, including China and India [14,18].

The major sources of antibiotic residues in milk include overdosing, failure to comply with recommended withdrawal periods, use of contaminated water, and improper disposal of animal waste [19,20]. Importantly, even appropriate therapeutic use can result in residues that pose risks to consumers [21]. Of particular concern is the occasional use of certain antibiotics classified as critically important for human medicine (CIAs) in mastitis treatment, along with the potential for cross-resistance to CIAs due to selective pressure from the widespread use of other veterinary antimicrobials, representing significant One Health challenges at the intersection of animal welfare and public health [22].

The key risks include the emergence of antibiotic-resistant bacteria and adverse effects on consumer health. Resistant strains can spread via food, including dairy products, complicating treatment of human infections [19,23]. High-density farming environments further promote resistance, as AMR genes and bacteria accumulate in manure, soil, and water [24,25]. Consumption of sub-inhibitory concentrations of antibiotic residues, whether in raw or cooked foods, can place selective pressure on human microbiota, promoting AMR persistence [26]. Such exposures enhance horizontal gene transfer, particularly plasmid-mediated conjugation, thereby facilitating the spread of resistance genes [27,28,29]. They may also impair immune function, posing particular risks to vulnerable groups, such as infants and immunocompromised individuals. Increasing recognition of the resistance selection potential of low-level residues underscores their contribution to pathogen transmission and the global AMR crisis, reinforcing the need for coordinated control strategies [30,31].

Considering the points discussed, the use of antibiotics in mastitis therapy and the resulting residues in milk represent one of the most pressing challenges in dairy farming today. Therefore, this review aims to provide a comprehensive synthesis of the current knowledge on mastitis therapeutic management, antibiotic use and the occurrence of antibiotic residues in bovine milk and dairy by-products during the period 2010–2022. Specifically, it summarizes available data on the frequency and types of detected antimicrobials, evaluates compliance with maximum residue limits (MRLs), and discusses the broader implications for animal welfare, public health, and environmental safety within a One Health–One Welfare framework. In addition, the review highlights alternative and sustainable strategies that can reduce antibiotic dependence while controlling udder health and milk quality.

## 2. Literature Search Strategy

This narrative review is based on a comprehensive literature search conducted in the PubMed, Scopus, and Web of Science databases, supplemented by official monitoring reports of antibiotic residues in milk submitted annually by EU Member States to the European Commission (DG SANTE/EFSA). Keywords used in various combinations included: antibiotic residues, milk contamination, bovine milk, human health, microbiota, antimicrobial resistance, immunological effects, and public health risk. In addition, terms related to animal welfare were included, such as: animal welfare, mastitis pain, dairy cow behavior, welfare assessment, and One Health/One Welfare. The search covered literature published between 2010 and 2024, with a focus on studies written in English. Preference was given to peer-reviewed articles, systematic reviews, and regulatory reports from international health agencies such as the World Health Organization (WHO), The European Food Safety Authority (EFSA), and Food and Agriculture Organization (FAO). Additional relevant sources were identified through manual screening of reference lists. Studies were selected for inclusion based on their relevance to the topic of antibiotic residue exposure through dairy consumption, including but not limited to their microbiological and immunological effects, to provide a balanced overview of the literature, as well as their contribution to understanding the welfare implications of mastitis and antimicrobial use in dairy cattle. Studies were excluded if they were not written in English, lacked relevance to antibiotic residues or animal welfare aspects, or did not provide primary or review data (e.g., conference abstracts, editorials, or opinion papers). Duplicate publications and studies with insufficient methodological details were also excluded.

## 3. Epidemiology and Impact of Bovine Mastitis

Bovine mastitis is among the most prevalent diseases in dairy cattle worldwide, with estimates indicating that up to 40% of cows in a herd may be affected annually, depending on management practices, region, and production system [32,33,34]. Generally, the annual incidence of clinical mastitis in well-managed dairy herds ranges between 20% and 40%, while subclinical mastitis may affect 25–65% of cows, with higher rates observed in intensive production systems [32,33,34].

This disease substantially affects animal health and welfare, impacts both milk production and reproductive efficiency, and raises public health concerns due to zoonotic pathogens, and the development of AMR [35]. Economically, mastitis imposes a substantial burden on the dairy industry worldwide. These losses are associated with decreased milk yield, discarded or contaminated milk, treatment costs, increased labor, veterinary care, and the premature removal of affected cows from the herd [36,37].

In addition to financial losses, mastitis poses serious animal welfare concerns [38]. Affected cows often experience pain, swelling of the udder, fever, and reduced feed intake, which can negatively impact overall health and productivity [39]. Chronic or recurrent mastitis can lead to prolonged discomfort, stress, and susceptibility to secondary infections, ultimately resulting in early culling and reduced productive period for the herd [7]. Behavioral changes such as reduced grooming, less time lying comfortably, and increased restlessness have been consistently observed in mastitic cows [7]. Elevated stress markers, including cortisol, further highlight the compromised welfare status [40]. Frequent antibiotic treatments may also cause additional handling stress and potential side effects, thereby indirectly affecting welfare [41]. Recognizing mastitis as both a health and a welfare problem emphasizes the need for multidimensional monitoring and management approaches.

Clinically, mastitis is characterized by inflammation of the udder and mammary tissues, which may result from trauma, chemical irritation, or, most commonly, infection by microorganisms such as fungi, viruses, algae, and especially bacteria [36]. Among these etiological agents, bacterial intramammary infections are considered the predominant cause. A wide spectrum of bacterial pathogens is implicated in mastitis, generally classified as either contagious or environmental. Major contagious pathogens, such as *Staphylococcus aureus*, *Streptococcus agalactiae*, and *Mycoplasma bovis* are primarily transmitted during the milking process [33,42]. In contrast, environmental pathogens, including *Escherichia coli*, *Streptococcus uberis*, and *Klebsiella* spp., and coagulase-negative staphylococci originate from bedding, soil, and manure [42,43]. The diversity, adaptability, and pathogenic complexity of these microorganisms pose major challenges for both the prevention and effective treatment of the disease.

Mastitis profoundly affects both milk yield and its technological quality [44]. Infected cows can produce 10–25% less milk per lactation, with chronic or recurrent infections resulting in even greater cumulative losses over time [45]. The disease also induces significant alterations in milk composition, including elevated somatic cell counts, increased levels of proteolytic enzymes such as plasmin, and reductions in lactose, casein, and fat content [35,46]. These compositional changes compromise the milk’s suitability for processing into cheese, yogurt, and other dairy products, thereby amplifying economic losses for producers. Furthermore, the altered biochemical properties of milk can affect shelf life and sensory characteristics, posing additional challenges for the dairy industry [47,48].

The public health implications of mastitis should also be highlighted, as milk from infected udders can pose a risk to human health due to the presence of pathogenic microorganisms and their toxins. In particular, mastitis caused by *S. aureus* carries the potential for enterotoxin contamination in milk and dairy products [49]. It is estimated that approximately 62% of isolated mastitis pathogens exhibit resistance to at least one antibiotic, and some of these pathogens are zoonotic [50]. Furthermore, due to risk of zoonotic transmission, milk from affected cows is unsuitable for consumption or sale, further contributing to substantial economic losses [51].

## 4. Antibiotic Therapy in Mastitis Management

In mastitis management, the primary aim of antibiotic therapy is to eliminate the causative pathogens from the mammary gland [5]. Therapy is typically administered intramammarily or parenterally, with intramammary administration being more common, as it results in higher local concentrations of drug achieved by lower doses [9]. The most commonly used antibacterial drugs are antibiotics, including cephalosporins, penicillin, erythromycin, quinolones, and sulfonamides [46,52]. During lactation, only clinical mastitis and certain subclinical forms are generally treated, as subclinical mastitis has lower cure rates and economic justification is limited [53,54]. Immediate therapy for clinical cases is recommended based on clinical signs, while milk samples should ideally be collected to identify pathogens and guide treatment if initial therapy is ineffective [53]. Additionally, dry cow therapy is commonly employed at the end of lactation to prevent the occurrence of new infections and reduce the incidence of clinical mastitis in the subsequent lactation [55].

The need for antibiotic therapy depends on the etiology of infection and the cow’s immune response [56]. In many cases, pathogens are not detected in milk samples, making antibiotic use difficult to justify. According to current EU legislation, bacteriological testing of milk samples is recommended before initiating antibiotic treatment, whenever practical, to ensure targeted and prudent antimicrobial use. However, in routine farm conditions, farmers often base treatment decisions on observed clinical symptoms without microbiological confirmation of infection, which can lead to inappropriate or ineffective therapy [57]. Even with proper antibiotic treatment, bacteriological cure rates rarely exceed 60%, and for *S. aureus*, effectiveness may be as low as 15% [58,59]. Post-treatment, inflammation can persist, and somatic cell counts may remain elevated, increasing the risk of recurrence [46].

Limitations of antibiotic therapy include the development of AMR, food safety concerns, high costs, and unfavorable withdrawal periods [60]. Milk from cows treated intramammarily often contains antibiotic residues and must be discarded until the withdrawal period is complete, creating significant economic losses. Antibiotic residues can cause adverse health effects, including hypersensitivity reactions, disturbances of the gut microbiome, and potential toxicity. They may also interfere with dairy processing by inhibiting starter cultures, affecting product quality and production efficiency [5,9,61]. Environmental contamination is another concern, as a large proportion of administered antibiotics is excreted unchanged into soil and water, potentially impacting microbial ecosystems and food safety [60]. The extent of environmental contamination largely depends on the route of administration. Systemic treatments, such as intramuscular or intravenous injections, result in widespread distribution of the drug throughout the animal’s body, with significant fractions excreted via urine and feces into the environment [62,63]. In contrast, intramammary administration targets the infected udder directly, leading to more localized exposure and reduced systemic absorption, although discarded milk can still represent a source of environmental contamination.

## 5. Antibiotic Residues in Bovine Milk

### 5.1. Sources and Persistence of Antibiotic Residues in Milk

Residues are defined by the European Medicines Agency (EMA) as pharmacologically active substances (including their metabolites and degradation products) that remain in foodstuffs obtained from treated animals [64]. The safety of these residues in food is described by Acceptable Daily Intake (ADI), which represents the amount that can be consumed daily over a lifetime without posing a toxicological or health-related risk to humans [65]. EMA worked closely with the EFSA to ensure a common approach on assessing human dietary exposure to residues in food from animal origin. Specific guidance on establishing withdrawal periods for milk aimed at preventing violative residues is provided in EMA’s dedicated guideline [66]. Actually, regulatory agencies such as EMA [66], the Codex Alimentarius Commission (FAO/WHO) [67], and national authorities such as the United States Food and Drug Administration (FDA), have established maximum residue limits (MRLs) for veterinary drugs to ensure that food produced from treated animals is safe for human consumption, both in the short and long term. In addition to existing EMA and Codex guidelines, the European Regulation 2019/6 on veterinary medicinal products provides a comprehensive legal framework governing the use of antibiotics in food-producing animals within the EU. This regulation sets out requirements for the authorization, prescription, and administration of veterinary drugs, including mandatory adherence to withdrawal periods to prevent residues in milk and other animal-derived foods. By establishing clear obligations for responsible antimicrobial use, Regulation 2019/6 aims to protect consumer health, minimize the risk of antimicrobial resistance, and ensure that milk placed on the market complies with established maximum residue limits (MRLs) [64]. Actually, MRLs have been established for antibiotics in milk to ensure that any antibiotic residues present do not pose a health risk to consumers [2]. It is important to highlight that milk intended for human consumption must not exceed these established MRLs [68].

Although both EMA and the Codex Alimentarius Commission establish MRLs to ensure food safety, these values may differ due to variations in regional regulatory frameworks, risk assessment approaches, and updates, with EMA MRLs being legally binding within the EU, while Codex MRLs serve as internationally recognized reference standards adopted by multiple countries worldwide.

In Table 1 are summarized CIAs for human medicine (as classified by WHO) [69] alongside their corresponding MRLs in milk (where established) and ADIs given by different authorities. Of particular concern are critically important antimicrobials of the highest priority (HP-CIAs), such as fluoroquinolones (e.g., ciprofloxacin), third- and fourth-generation cephalosporins (e.g., ceftriaxone, cefepime), polymyxins (e.g., colistin), and glycopeptides (e.g., vancomycin), for which MRLs are often not established or their use in food-producing animals is restricted or prohibited due to the high risk of transferring resistance genes to human pathogens. For example, no MRLs exist for meropenem, vancomycin, linezolid, and tigecycline, reflecting their critical importance in treating multidrug-resistant infections in humans and the lack of authorization for their use in food-producing animals. By contrast, for some lower-risk CIAs, such as ampicillin, gentamicin, and erythromycin, MRLs have been established by the EU and Codex [66,67], and corresponding ADI values have been determined by EMA [70] EFSA [71] and Joint FAO/WHO Expert Committee on Food Additives (JECFA) [72] based on toxicological data.

Antibiotic residues in milk primarily result from the improper use of antibiotics in treating animal infections, with key factors including dry cow therapy and the treatment of mastitis, endometritis, and respiratory issues [15,73]. While most drugs are metabolized and eliminated through urine and, to a lesser extent, feces, some may also persist in milk, eggs, and meat. This is especially alarming since significant amounts (30–70%) of antibiotics may remain unchanged in animals’ organs and tissues, potentially retaining pharmacological activity [15].

The main antibiotic classes used in dairy cows include beta-lactams, chloramphenicols, macrolides, quinolones, sulfonamides, aminoglycosides, and tetracyclines [45,46]. Among these, beta-lactams are predominant. Sachi et al. [15] reported that penicillins accounted for 75% of use and cephalosporins for 43.61%, while aminoglycosides represented only 10.44%. A 2021 systematic review from China showed a similar trend, with beta-lactams being the most frequently detected residues in milk, followed by tetracyclines (tetracycline and oxytetracycline) and sulfonamides [74]. This highlights growing concerns about the increasing use of beta-lactam antibiotics in dairy farming which has historically have been the primary choice for mastitis treatment, predominantly administered Via intramammary infusion due to their broad-spectrum antibacterial activity, which supports their frequent detection in residue studies [75]. The growing use of beta-lactams in dairy farming is particularly concerning, as some of these antibiotics, such as penicillins and cephalosporins, are classified by the WHO as CIAs for human health, making their widespread use in animals a potential threat to public health [76]. Feeding calves with milk from cows treated during lactation is an inappropriate management practice, as it can lead to increased fecal shedding of antimicrobial-resistant bacteria and contribute to the spread of resistance [77].

The occurrence of antibiotic residues in milk is closely linked to the rigor of regulatory control measures, which tend to be stricter in developed countries. This is supported by the results of a comprehensive study performed by Costa et al. [75], which reported the lowest incidence of antibiotic residues in Italy (0.09%), and the highest in Tanzania (82.70%). Actually, tetracyclines were the most frequently detected antibiotic group (43.75%), followed by beta-lactams and sulfonamides (31.25%), and quinolones (25%).

Additional studies provide a broader global perspective. In Iran, approximately 29% of dairy products contained antibiotic residues, with tetracyclines showing the highest levels and contamination varying by province, highlighting the need for nationwide monitoring and stronger regulatory oversight [1]. In Kenya, 24% of milk from vending machines and street vendors contained at least one antibiotic residue, mainly tetracycline and gentamicin [78]. In Brazil, 17% of pasteurized milk samples tested positive for β-lactam or tetracycline residues, with 11 samples positive for β-lactams and 6 for both β-lactams and tetracyclines [79]. In India, 10.23% of pooled raw milk samples contained antibiotic residues, with 2.33% positive for oxytetracycline and 1.86% exceeding the maximum residue limit [80]. Similarly, in Pakistan, over 50% of raw milk samples exceeded the maximum residue limit for tetracycline, indicating a significant risk of antimicrobial exposure through food [81].

### 5.2. Monitoring of Antibiotic Residues in Milk

It has been reported that developing countries are at a higher risk regarding frequency of antibiotic residues presence in milk compared to developed nations, largely due to limited detection capabilities and inadequate residue monitoring systems, which often fail to enforce compliance with recommended MRLs [15]. Consequently, the worst-case scenario is seen in many low- and middle-income countries, where regulatory frameworks and surveillance are minimal or non-existent, leading to the indiscriminate sale and widespread use of veterinary antimicrobials [82].

Efficient monitoring of antibiotic residues in food matrices relies on various diagnostic techniques. Ideally, a test should be capable of identifying a broad spectrum of antibiotic residues, be quick to perform, and cost-effective. The existing methods for the detection of antibiotic residues differ in terms of accuracy, speed, and cost [73]. Modern approaches combine screening and confirmatory methods [83,84]. Moreover, for rapid screening of antibiotics in milk, microbiological tests, immunoassays, and biosensors are commonly used. Here, microbiological methods refer to bioassays that detect antibiotic residues based on their inhibitory effects on the growth of specific indicator microorganisms. These methods are often preferred due to their convenience, low cost, and broad-spectrum capabilities. They can be performed by non-professionals, and are usually quick, and cost-effective [83,85,86]. Enzyme-linked immunosorbent assays and fluorescence immunoassays are simple, eco-friendly and cost-effective screening tools. Furthermore, electrochemical sensors have revolutionized modern analysis with their simplicity and rapid response, directly transmitting data to electronic equipment. However, the electrode preparation process can be complex, and some electrodes require modification with expensive materials [85]. Despite these advantages, screening techniques have limited sensitivity and specificity, leading to potential false positives or false negatives [87].

Confirmatory methods, typically based on liquid chromatography–tandem mass spectrometry (LC-MS/MS), are considered the gold standard for monitoring antibiotics in food [87,88]. Their main advantages include high specificity and sensitivity, potential for automation, and rapid turnaround time [86,87]. However, these methods require complex sample preparation, consume large quantities of consumables, and are costly to operate. Additionally, the use of significant amounts of toxic organic solvents raises environmental concerns [88].

Significant progress has been made over the past decade in developing analytical methods for detecting different antibiotic groups in various raw food matrices, such as milk, meat, and eggs. However, method development for processed dairy products remains limited, despite evidence that antibiotics are not completely degraded during pasteurization [89].

### 5.3. Occurrence of Antibiotic Residues in Bovine Milk and Dairy By-Products (2010–2022)

Across the 2010–2022 European dataset summarized in Table 2, the proportion of non-compliant samples remained below 1% in most countries, with β-lactams being the most frequently detected antimicrobial class. These results confirm the effectiveness of EU residue-monitoring programs, while isolated non-compliant findings—mainly under suspect sampling—indicate occasional lapses in withdrawal compliance. The summarized data derive from official annual residue monitoring reports submitted by EU Member States to the European Commission (DG SANTE/EFSA) for the period 2010–2022, covering both targeted and suspect sampling across 24 European countries. Overall, the consistently low rate of non-compliance reflects the success of residue surveillance and improved antimicrobial stewardship in dairy production, yet the recurrent detection of β-lactams and tetracyclines underscores the need for continued monitoring and farmer education.

## 6. Public Health Implications

The human microbiota is essential for maintaining overall health, acting as a protective barrier against pathogens, aiding digestion, and supporting immune function. Approximately 95% of these microbes are beneficial bacteria, while the rest consist of harmful or opportunistic pathogens, together maintaining a stable equilibrium with the human body over time [103].

Antibiotic residues in milk can alter the intestinal microbiota of consumers, reducing beneficial bacteria and allowing resistant strains to thrive, a condition known as intestinal dysbiosis [104,105,106,107,108]. Even sub-therapeutic levels of residues may select for antibiotic-resistant bacteria, representing a direct public health concern and contributing to the global spread of antimicrobial resistance. These effects highlight the critical role of milk safety in the One Health framework [66,109].

Moreover, exposure to sub-therapeutic levels of antibiotics allows bacteria to adapt and evolve, leading to the emergence of antibiotic-resistant strains [107].

One of the most critical risks associated with antibiotic residues is the transfer of resistant bacteria to humans. The transfer may occur through the food chain and/or animal handlers [16]. AMR has far-reaching consequences, including prolonged hospitalizations, treatment failures, and increased mortality due to reduced antibiotic efficacy [110]. Managing resistant infections requires alternative treatments, often more expensive and less accessible, further endangering human health [111].

The presence of antibiotic residues in the milk can disrupt multiple physiological systems, including the immune, endocrine, nervous, and reproductive systems, and may contribute to the development of congenital anomalies [82]. Among the most commonly reported adverse effects are allergies, with infants, who primarily consume milk, being particularly vulnerable [21,73]. Furthermore, these immunological responses can range from mild rashes to life-threatening anaphylactic shock [112]. Most documented cases of antibiotic hypersensitivity involve penicillins, aminoglycosides, and tetracyclines [16]. Although β-lactam antibiotics are generally considered less toxic, they account for the majority of reported allergic reactions to antimicrobials in humans, with approximately 10% of the population being allergic to penicillin [107]. Reactions linked to β-lactam residues in milk include serum sickness, erythema multiforme, hemolytic anemia, thrombocytopenia, vasculitis, acute interstitial nephritis, Stevens-Johnson syndrome, and toxic epidermal necrolysis [112].

The long-term health effects of antibiotic residues remain largely unknown [16]. However, reports suggest that prolonged exposure to veterinary drug residues can result in both acute and chronic poisoning, including teratogenic, carcinogenic, and mutagenic effects [82]. For instance, chloramphenicol residues have been associated with an increased cancer risk and, even at low concentrations, can cause aplastic anemia [107]. Antibiotic residues may also trigger toxicological symptoms, including headaches, nausea, and diarrhea [54]. Interestingly, studies have linked the consumption of milk containing tetracycline, nitrofuran, and sulfonamide residues to conditions such as leukocytosis, lung congestion, atypical lymphocytes, thrombocytopenic purpura, toxic granulation of granulocytes, and tooth discoloration [112].

Beyond health concerns, antibiotic residues in milk also pose significant economic challenges for the dairy industry. The presence of antibiotic-resistant bacteria in the milk disrupts essential fermentation processes required for producing dairy products such as cheese, yogurt, and kefir. These microorganisms can interfere with fermentation, decrease acid formation, reduce the curdling of milk and cause an improper ripening of cheeses, ultimately compromising product quality, taste, and safety [83,86]. By interfering with fermentation, they extend production times and negatively impact the sensory characteristics of dairy products, leading to financial losses [88].

## 7. Environmental and Food Chain Considerations

Antibiotics administered for the treatment of bovine mastitis are not fully metabolized in the animal and are partly excreted through urine and feces. These residues can accumulate in manure and slurry, which are often applied to agricultural land or discharged into water systems. Such practices contribute to the dissemination of antimicrobial residues in soil and aquatic environments, where they exert selective pressure on microbial communities and promote the emergence and persistence of resistant bacteria. Mobile genetic elements further facilitate the transfer of resistance determinants to pathogenic species, posing risks for both animal and human health [113].

Contamination of the food chain represents another critical concern. Inadequate adherence to withdrawal periods may result in antibiotic residues persisting in raw milk, which can reach consumers directly, or through processed dairy products. Although certain processing methods reduce residue concentrations, they do not eliminate them completely [75,107]. In addition to monitoring antibiotic residues directly, somatic cell count and differential cell count can serve as valuable indicators of udder health and subclinical mastitis, helping to identify animals at risk and optimize treatment strategies [114].

Beyond direct exposure, residues entering the environment may bioaccumulate in plants, aquatic organisms, and soil ecosystems, introducing additional pathways of human exposure through the food chain [115]. This ecological impact highlights the interconnectedness of animal production systems, environmental health, and food safety. Effective mitigation strategies include the enforcement of withdrawal periods, adoption of residue monitoring and detection programs across the dairy supply chain, and sustainable manure management practices such as composting or anaerobic digestion to reduce the antimicrobial load. Strengthening farm-to-fork surveillance within a One Health framework is essential to limit the environmental and food chain dissemination of antibiotic residues and resistant bacteria [116,117].

## 8. Alternative Approaches for Prudent Antibiotic Administration in Dairy Farming

Prudent antibiotic use in livestock is a cornerstone of antimicrobial stewardship and a key element of the One Health approach [118]. While antibiotics remain indispensable for animal health and welfare, their use must be carefully managed to minimize the emergence of antimicrobial resistance and prevent residues in milk [119]. In response to these challenges, sustainable and evidence-based alternatives to conventional antibiotic therapy are being increasingly explored in dairy farming. These include biosecurity measures, vaccines, probiotics, and phytotherapy, as well as emerging technologies such as phage therapy and nanotechnology, which aim to reduce dependence on antibiotics while maintaining animal welfare and productivity [120,121].

In response to the growing concerns about antibiotic residues in bovine milk and their potential microbiological and immunological consequences for human health, there is an urgent need to explore sustainable alternatives to conventional antibiotic use in dairy farming. Non-antibiotic approaches eliminate residue problems and antibiotic resistance, and have become a topic of interest for public health and for research [122]. Several strategies have emerged as promising approaches to mitigate antibiotic dependence such as biosecurity, vaccines, probiotics and phytotherapy. Emerging technologies such as phage therapy and nanotechnology have not yet been fully integrated into mastitis management.

Farm biosecurity is a cost-effective approach to preventing infectious diseases in livestock by limiting the introduction and spread of pathogens through practices like quarantine, restricted movements, disinfection, and proper milking hygiene, thereby reducing the need for antibiotic use and the risk of AMR [123]. However, the adoption of these measures varies widely due to factors such as farmers’ perceptions, socioeconomic constraints, and limited evidence on their impact on antimicrobial usage (AMU), highlighting the need for regional and national assessments of biosecurity practices and their relationship to AMU to strengthen trust among stakeholders [124,125].

Phytotherapy, using plant-derived compounds like essential oils (EOs), is a promising alternative for preventing and treating bovine mastitis due to its antimicrobial, anti-inflammatory, and immunomodulatory effects, low risk of residues, low risk of adverse effects, low manufacturing costs, and potential to combat antibiotic resistance. While many In Vitro studies show EOs’ efficacy against mastitis pathogens [73,74,75,76,77,78,79], limited In Vivo results vary [126,127,128,129], though some EO-based products have demonstrated comparable effects to conventional treatments with added economic benefits, thus highlighting the need for further research in order to fully evaluate their potential in mastitis management. Study conducted by Kovačević et al. [130] reported shorter withdrawn period for EO-based formulation in treating bovine mastitis.

Probiotics, live microorganisms that promote host health by colonizing various body sites, show promise for mastitis prevention and treatment in dairy cows, with studies demonstrating some efficacy comparable to antibiotics and antimicrobial effects against *S. aureus* [131]. However, while early results are encouraging, these approaches require further rigorous validation in animal models and larger field trials to confirm their safety and effectiveness as part of integrated mastitis control strategies [131,132].

Advances in immunology have led to the development of vaccines that enhance the cow’s natural defenses against common pathogens. These alternative strategies not only reduce the risk of antibiotic residues in milk but also support antimicrobial stewardship and align with global efforts to combat antibiotic resistance. Despite decades of research, an effective vaccine for preventing bovine mastitis remains elusive due to challenges like limited efficacy, strain diversity, and varying environmental factors, though some vaccines such as those targeting *S. aureus* and *E. coli* show partial protection and ongoing research aims to develop more broadly protective solutions [133]. Vaccines targeting *S. aureus* and *E. coli* aim to boost immune responses in dairy cows, reduce antibiotic use, and improve resistance to mastitis, though their efficacy varies due to differences in vaccine design, cow immunity, and farm conditions. Despite commercial availability of some vaccines, their limited coverage of diverse pathogens highlights the need for future research on broader-spectrum vaccines, improved delivery methods, and integration with non-antibiotic therapies for effective mastitis control [131]. While some vaccines show promising potential, results of the conducted studies are inconsistent, and vaccination alone is usually insufficient without integration into broader antimicrobial stewardship and control programs in order to effectively combat mastitis and AMR [6].

## 9. One Health-One Welfare Perspective

Mastitis exemplifies the interconnectedness of animal health, welfare, public health, and the environment. As mentioned above, from a One Health perspective, the overuse of antibiotics contributes to AMR and the presence of residues in milk, posing risks to human health. From a One Welfare perspective, untreated or poorly managed mastitis undermines animal well-being, increasing stress and suffering. In addition, environmental contamination with antibiotic residues and resistant bacteria creates further risks for ecosystems [134,135]. The importance of linking animal welfare with global sustainability goals has been emphasized in recent discussions on the United Nations Sustainable Development Goals (SDGs), where experts concluded that although welfare is not explicitly mentioned, progress toward the SDGs is compatible with advancing animal welfare [136]. The concept of One Welfare has been proposed as a complement to the One Health approach [137,138]. By strengthening the connection between science, policy, and sustainability, it highlights the interdependence of human, animal, and environmental welfare [8]. Addressing mastitis therefore requires integrated approaches that simultaneously safeguard animal welfare, public health, and environmental sustainability.

## 10. Conclusions

Antibiotic therapy remains a cornerstone of mastitis management, supporting animal welfare and herd productivity. However, the persistence of antibiotic residues in milk presents significant public health challenges, including AMR and potential immunological effects in humans. Effective monitoring, adherence to withdrawal periods, and responsible antibiotic use are critical to minimizing these risks. Sustainable alternatives, such as probiotics, phytotherapy, vaccines, and improved biosecurity, offer promising strategies to reduce antibiotic dependence. A holistic One Health–One Welfare approach, linking animal welfare, human health, and environmental sustainability, is essential for the future of safe and responsible dairy farming.

## Figures and Tables

**Table 1:** Maximum residue limits (MRL) in milk and acceptable daily intake (ADI) for critically important antimicrobials (CIAs) for human use.

Antibiotic Class & Active Substance	WHO Category	EU MRL in Milk (µg/kg or µg/L)	Codex MRL in Milk (µg/kg or µg/L)	ADI (mg/kg bw/day)
Aminoglycosides: Gentamicin	HP-CIA	100 µg/L	200 µg/L	0.1 (EFSA) 0.02 (JECFA)
Ansamycins: Rifampicin	HP-CIA	no MRL	—	0.03 (JECFA)
Carbapenems: Meropenem	HP-CIA	no MRL	—	not established
Cephalosporins (3rd–5th gen): Ceftiofur	HP-CIA	100 µg/L	100 µg/L	0.01 (EFSA) 0.05 (JECFA)
Cephalosporins: Ceftriaxone, Cefepime	HP-CIA	no MRL	—	not established
Glycopeptides: Vancomycin	HP-CIA	no MRL	—	not established
Glycylcyclines: Tigecycline	HP-CIA	no MRL	—	not established
Lipopeptides: Daptomycin	HP-CIA	no MRL	—	not established
Macrolides: Azithromycin, Erythromycin	HP-CIA	40 µg/kg (Erythromycin)	—	0.2 (EFSA for erythromycin) 0.07 (JECFA for erythromycin)
Monobactams: Aztreonam	HP-CIA	no MRL	—	not established
Oxazolidinones: Linezolid	HP-CIA	no MRL	—	not established
Penicillins (antipseudomonal): Piperacillin	HP-CIA	no MRL	—	not established
Penicillins (aminopenicillins): Ampicillin, Amoxicillin-clavulanic acid	CIA	4 µg/kg	4 µg/kg	0.3 (EFSA)
Polymyxins: Colistin	HP-CIA	50 μg/kg	—	7 µg/kg bw/day (JECFA)
Quinolones: Ciprofloxacin	HP-CIA	100 μg/kg	—	0.03 (EFSA)
Tuberculosis drugs: Isoniazid	HP-CIA	no MRL	—	0.01 (JECFA)

**Table 2:** The occurrence of non-compliant results in milk samples analyzed for antibiotics published by EFSA for period 2010–2022; N_t_—total number of analyzed samples, N_nC_—number of non-compliant results, countries: DE—Germany, BE—Belgium, CY—Cyprus, FR—France, FI—Finland, LT—Lithuania, CZ—The Czech Republic, ES—Spain, PL—Poland, IT—Italy, SK—Slovakia, UK—The United Kingdom, IE—Ireland, SI—Slovenia, LU—Luxemburg, HR—Croatia, AT—Austria, DK—Denmark, FI—Finland, N—Norway, EE—Estonia, RO—Romania, LV—Latvia, MT—Malta.

Year	Sampling Antibiotic	Targeted Sampling					Suspect Sampling			
		Country	N_t_	N_nC_	N_nC_ (%)	Antibiotic	Country	N_t_	N_nC_	N_nC_ (%)
2010 [90]	Ampicillin	DE	340	1	0.29	Ampicillin	IT	165	1	0.61
	Cloxacillin	BE	143	1	0.70	Penicillin G	IT	165	1	0.61
	Inhibitors	CY	3000	12	0.40	Cloxacillin	DE	13	3	23.08
	Tetracycline	FR	330	1	0.30	Oxytetracycline	IT	164	1	0.61
2011 [91]	Penicillin G	FI	220	1	0.45	Ampicillin	IT	224	1	0.45
	Penicillin G	LT	211	1	0.47					
	Cefoperazon	CZ	1	1	100.00					
	Doxycycline	ES	471	1	0.21					
	Inhibitors	CY	2600	8	0.31					
	Tetracycline	PL	1797	2	0.11					
2012 [92]	Ampicillin	LT	214	1	0.47	Penicillin G	IT	52	3	5.77
	Penicillin G	DE	404	1	0.25	Oxytetracycline	IT	41	1	2.44
	Penicillin G	IT	112	1	0.89	Spiramycin	IT	15	2	13.33
	Penicillin G	SK	45	1	2.22					
	Cefalonium	FR	327	1	0.31					
	Inhibitors	CY	3131	3	0.10					
	Oxytetracycline	LT	214	1	0.47					
2013 [93]	Amoxycillin	PL	1783	1	0.06	Penicillin G	DE	11	1	9.09
	Amoxycillin	UK	1646	1	0.06	Oxytetracycline	DE	123	1	0.81
	Ampicillin	CY	2920	1	0.03	Oxytetracycline	IT	140	1	0.71
	Penicillin G	DE	445	1	0.22					
	Penicillin G	PL	1783	1	0.06					
	Cloxacillin	IE	312	1	0.32					
	Cloxacillin	SI	144	1	0.69					
	Doxycycline	ES	141	3	2.13					
	Inhibitors	CY	2920	4	0.14					
	Penicillin	UK	1646	2	0.12					
2014 [94]	Amoxycillin	FR	301	1	0.33	Penicillin G	AT	1	1	100.00
	Ampicillin	LU	55	1	1.82	Penicillin G	IT	82	2	2.44
	Penicillin G	PL	99	1	1.01					
	Cefalonium	FR	301	1	0.33					
	Dihydrostreptomycin	UK	1517	1	0.07					
	Doxycycline	PL	99	2	2.02					
	Inhibitors	CY	3027	12	0.40					
	Spiramycin	ES	319	1	0.31					
2015 [95]	Amoxycillin	CY	71	1	1.41	Amoxycillin	IT	103	1	0.97
	Amoxycillin	UK	474	1	0.21	Ampicillin	ES	21	2	9.52
	Penicillin G	IT	147	1	0.68	Ampicillin	GR	6	1	16.67
	Cefalexin	DE	311	1	0.32	Penicillin G	IT	103	1	0.97
	Cefquinom	IT	29	1	3.45	Cloxacillin	ES	21	2	9.52
	Cloxacillin	PL	120	1	0.83	Cloxacillin	IT	103	1	0.97
	Enrofloxacin	ES	381	1	0.26	Tilmicosin	IT	100	2	2.00
	Enrofloxacin	PL	120	1	0.83					
	Kanamycin	DE	35	1	2.86					
	Spiramycin	ES	313	1	0.32					
	Tetracycline	PL	120	1	0.83					
2016 [96]	Amoxycillin	UK	481	1	0.21	Cefalonium	IT	13	1	7.69
	Penicillin G	DE	509	1	0.20	Cefazolin	IT	13	1	7.69
	Danofloxacin	ES	281	1	0.36	Cloxacillin	DE	13	1	7.69
	Gentamicin	PL	140	1	0.71	Gentamicin	PL	2	1	50.00
	Tetracycline	PL	140	2	1.43	Lincomycin	IT	1	1	100.00
	Tilmicosin	IT	96	1	1.04	Na-penicillin-G	ES	1	1	100.00
						Tilmicosin	IT	79	1	1.27
2017 [97]	Ampicillin	HR	293	1	0.34	Cloxacillin	IT	66	1	1.52
	Ampicillin	IT	274	1	0.36	Florfenicol	UK	6	2	33.33
	Penicillin G	ES	232	1	0.43	Tilmicosin	IT	67	1	1.49
	Penicillin G	UK	504	2	0.40					
	Cefalonium	IT	119	1	0.84					
	Ciprofloxacin	ES	288	1	0.35					
	Cloxacillin	HR	293	1	0.34					
	Cloxacillin	IT	270	1	0.37					
	Doxycycline	ES	273	2	0.73					
	Florfenicol	UK	869	5	0.58					
	Oxytetracycline	PL	1976	1	0.05					
	Tetracycline	PL	1976	1	0.05					
	Trimethoprim	AT	32	1	3.13					
	Tulathromycin	DK	211	1	0.47					
2018 [98]	Aminosidin	CY	83	1	1.20	Aminosidin	CY	1	1	100.00
	Amoxycillin	PL	1507	1	0.07	Penicillin G	DE	13	1	7.69
	Cloxacillin	IT	333	1	0.30	Penicillin G	IT	99	1	1.01
	Oxytetracycline	GR	150	1	0.67	Cloxacillin	DE	13	1	7.69
2019 [99]	Aminosidin	CY	87	1	1.15	Amoxycillin	IT	123	1	0.81
	Amoxycillin	IT	348	1	0.29	Penicillin G	DE	3	1	33.33
	Ampicillin	IT	422	1	0.24					
	Penicillin G	FI	244	1	0.41					
	Penicillin G	N	246	1	0.41					
	Penicillin G	RO	59	1	1.69					
	Dihydrostreptomycin	N	254	1	0.39					
	Doxycycline	PL	1717	1	0.06					
	Rifaximin	LV	211	1	0.47					
	Betalactams	RO	58	1	1.72					
	Tetracycline	PL	126	1	0.79					
2020 [100]	Ampicillin	LV	210	1	0.48	Cefalonium	IT	30	1	3.33
	Ampicillin	PL	1568	1	0.06	Cloxacillin	DE	3	1	33.33
	Penicillin G	FR	5	1	20.00					
	Penicillin G	DE	887	1	0.11					
	Penicillin G	N	229	1	0.44					
	Cefapirin	EE	177	1	0.56					
	Tetracycline	PL	1649	1	0.06					
2021 [101]	Amoxycillin	IT	339	1	0.29	Cefalonium	IT	26	1	3.85
	Amoxycillin	PL	1409	1	0.07	Tetracycline	FR	1	1	100.00
	Penicillin G	CY	85	1	1.18					
	Penicillin G	FI	304	1	0.33					
	Penicillin G	PL	1409	1	0.07					
	Cefquinom	LU	105	1	0.95					
	Tulathromycin	IT	174	1	0.57					
2022 [102]	Aminosidin	CY	83	1	1.20	Cloxacillin	AT	17	2	11.76
	Penicillin G	MT	240	1	0.42	Oxytetracycline	IT	65	1	1.54
	Penicillin G	PL	1905	1	0.05					
	Cloxacillin	HR	280	1	0.36					
	Florfenicol + metabolites	IT	190	1	0.53					

## Data Availability

No new data were created or analyzed in this study. Data sharing is not applicable to this article.

## Abbreviations

The following abbreviations are used in this manuscript:

ADI	Acceptable daily intake
AMR	Antimicrobial resistance
AMU	Antimicrobial usage
CIAs	Critically important antimicrobials
EFSA	The European Food Safety Authority
EMA	European Medicines Agency
EOs	Essential oils
EU	European Union
FAO	Food and Agriculture Organization
FDA	United States Food and Drug Administration
HP-CIAs	Critically important antimicrobials of the highest priority
JECFA	Joint FAO/WHO Expert Committee on Food Additives
MRLs	Maximum residue limits
WHO	World Health Organization
LC-MS-MS	Liquid Chromatography Tandem Mass Spectrometry
SDGs	United Nations Sustainable Development Goals
N_t_	total number of analyzed samples
N_nC_	number of non-compliant results
DE	Germany
BE	Belgium
CY	Cyprus
FR	France
FI	Finland
LT	Lithuania
CZ	The Czech Republic
ES	Spain
PL	Poland
IT	Italy
SK	Slovakia
UK	The United Kingdom
IE	Ireland
SI	Slovenia
LU	Luxemburg
HR	Croatia
AT	Austria
DK	Denmark
N	Norway
EE	Estonia
RO	Romania
LV	Latvia
MT	Malta
